# 

**Published:** 1846-07

**Authors:** 


### CONTENTS

OF NO. I.

### ORIGINAL COMMUNICATIONS.

ESSAYS AND CASES.

ART. I.—Cases of Amputation in Private Practice. By WILLIAM M. BOLING, M.D., of Montgomery, Alabama. - - - - - - - - - - 1

ART. II.—A Case of Epilepsy in a Boy, successfully treated. By Dr. THOMAS C. OSBORNE, of Erie, Ala. - - 16

REVIEWS.

ART. III.—Caloric, its Mechanical, Chemical, and Vital Agencies in the Phenomena of Nature. By SAMUEL L. METCALFE, M.D., of Transylvania University. - 19

ART. IV.—New Elements of Operative Surgery. By ALF. A. L. M. VELPEAU, Professor of Surgical Clinique of the Faculty of Medicine of Paris, Surgeon to the Hospital of La Charité, Member of the Royal Academy of Medicine, of the Institute, &c. Translated by P. S. TOWNSEND, M.D., late Physician to the Seamen's Retreat, Staten Island, New York. Under the supervision of, and with Notes and Observations by VALENTINE MOTT, M.D., Professor of the Operations of Surgery with Surgical and Pathological Anatomy, in the University of New York; Foreign Associate of the Académie Royale de Médecine of Paris, of that of Berlin, Brussels, Athens, &c. - - 50

SELECTIONS FROM AMERICAN AND FOREIGN JOURNALS.

Magendie's Introductory Lecture at the College of France. - 73
Prescription of Castor Oil. - - - - - - - - 78
Graves on the Relapse Periods of Ague, and the Indications of Treatment. - - - - - - - - - - 78
Remarkable Case of Paraphymosis. - - - - - - 79
Electro-Magnetism in a case of Obstruction of the Bowels. - 81
Medical Examinations in England. - - - - - - 82
Large Biliary Calculus. - - - - - - - - 82
The Asiatic Cholera. - - - - - - - - 83

ORIGINAL INTELLIGENCE.

Foreign Correspondence. - - - - - - - - - 85
Johnston's Natural Philosophy. - - - - - - - 89
To Correspondents. - - - - - - - - - 89
Books Received. - - - - - - - - - 89
Buffalo Medical Journal. - - - - - - - - 90
Commission Agency for Professional Business. - - - 90
Private Medical Teaching. - - - - - - - 89
Dictionary of Practical Medicine. - - - - - 91
Trismus Nascentium. - - - - - - - - 91
The Southern Journal of Medicine. - - - - - - 92
Medical Miscellany. - - - - - - - - - - 92

